# Sperm quality and paternal age: effect on blastocyst formation and pregnancy rates

**DOI:** 10.1186/s12610-016-0045-4

**Published:** 2017-01-21

**Authors:** Aurélie Chapuis, Anna Gala, Alice Ferrières-Hoa, Tiffany Mullet, Sophie Bringer-Deutsch, Emmanuelle Vintejoux, Antoine Torre, Samir Hamamah

**Affiliations:** 1 ART/PGD Division, CHU Montpellier, Montpellier, F-34295 France; 2 INSERM U1203, Montpellier, F-34295 France; 3 Montpellier University, UFR of Medicine, Montpellier, France; 4 Gynaecology and Obstetric Department, CHU Montpellier, Montpellier, F-34295 France

**Keywords:** Sperm quality, Sperm concentration, Progressive motility, Paternal age, Blastulation rate, Rate of clinical pregnancy, Qualité du sperme, Concentration spermatique, Mobilité progressive- age de l’homme- taux de blastulation, Taux de grossesses cliniques

## Abstract

**Background:**

Several studies suggest a decrease in sperm quality in men in the last decades. Therefore, the aim of this work was to assess the influence of male factors (sperm quality and paternal age) on the outcomes of conventional in vitro fertilization (IVF) and intracytoplasmic sperm injection (ICSI).

**Methods:**

This retrospective study included all couples who underwent IVF or ICSI at Montpellier University Hospital, France, between 1 January 2010 and 31 December 2015. Exclusion criteria were cycles using surgically retrieved sperm or frozen sperm, with pre-implantation genetic diagnosis or using frozen oocytes. The primary outcomes were the blastulation rate (number of blastocysts obtained at day 5 or day 6/number of embryos in prolonged culture at day 3) and the clinical pregnancy rate. The secondary outcomes were the fertilization and early miscarriage rates.

**Results:**

In total, 859 IVF and 1632 ICSI cycles were included in this study. The fertilization rate after ICSI was affected by oligospermia. Moreover, in ICSI, severe oligospermia (lower than 0.2 million/ml) led to a reduction of the blastulation rate. Reduced rapid progressive motility affected particularly IVF, with a decrease of the fertilization rate and number of embryos at day 2 when progressive motility was lower than 32%.

Paternal age also had a negative effect. Although it was difficult to eliminate the bias linked to the woman’s age, pregnancy rate was reduced in IVF and ICSI when the father was older than 51 and the mother older than 37 years.

**Conclusions:**

These results allow adjusting our strategies of fertilization technique and embryo transfer. In the case of severe oligospermia, transfer should be carried out at the cleaved embryo stage (day 2–3) due to the very low blastulation rate. When the man is older than 51 years, couples should be aware of the reduced success rate, especially if the woman is older than 37 years. Finally, promising research avenues should be explored, such as the quantification of free sperm DNA, to optimize the selection of male gametes.

## Résumé

**Contexte:**

De nombreuses données suggèrent une altération des paramètres spermatiques ces dernières décennies. Le but de ce travail est d’évaluer l’impact de facteurs masculins tels la qualité du sperme et l’âge paternel sur les résultats en fécondation in vitro classique (FIVc) et en fécondation in vitro avec injection intra-cytoplasmique de spermatozoïde (ICSI).

**Matériels et méthodes:**

L’étude a porté sur l’ensemble des couples ayant fait l’objet d’une tentative de FIVc ou d’ICSI entre le 1^er^ janvier 2010 et le 31 décembre 2014 au CHU de Montpellier.

Les critères d’exclusion ont été les tentatives avec utilisation de spermatozoïdes prélevés chirurgicalement ou de sperme congelé, les cycles avec diagnostic pré-implantatoire et les cycles avec ovocytes congelés.

Au total, 859 ponctions de FIVc et 1632 ponctions d’ICSI ont été incluses dans l’étude.

**Résultats:**

En ICSI, le taux de fécondation est affecté par l’oligospermie. Par ailleurs, une oligospermie extrême (inférieure à 0,2 M/ml) entraîne une diminution du taux de blastulation.

La mobilité progressive avant préparation a plus d’impact en FIVc, où les taux de fécondation et le nombre d’embryons obtenus à J2 vont être plus bas lorsque la mobilité progressive est inférieure à 32%.

Même s’il est difficile d’éliminer le biais lié à l’âge de la partenaire, il semblerait qu’il y ait une diminution du taux de grossesse en FIVc et en ICSI à partir de 51 ans chez l’homme avec une partenaire âgée de plus de 37 ans.

**Conclusion:**

Ces résultats permettront essentiellement d’ajuster nos stratégies de choix de technique de mise en fécondation et de transfert.

Pour les oligospermies extrêmes, il semble préférable de proposer un transfert précoce au stade embryon clivé (J2 - J3) car le taux de blastulation est très réduit dans ce cas.

Lorsque l’homme est âgé, il faudra également informer le couple de la diminution des taux de réussite, d’autant plus si sa partenaire a plus de 37 ans.

Enfin, différentes pistes prometteuses de recherche sont encore à explorer, comme le dosage de l’ADN libre spermatique afin d’optimiser la sélection des gamètes masculins et ainsi améliorer les résultats en AMP.

## Background

In France, 1 in 7 couples consult at some point for fertility problems [1]. In about 15% of couples, the infertility problem cannot be explained and in 30%, the origin is paternal [2]. Male infertility is diagnosed, among others, on the basis of quantitative or qualitative sperm anomalies visualized by semen analysis. The last guidelines by the World Health Organization (WHO) show a global decrease of the different sperm parameters compared with the previous edition of 1999 [3]. Concomitantly, several studies suggest a trend towards a reduced sperm quality in the last years [4].

Assisted reproduction technology (ART) is a therapeutic solution that allows circumventing human infertility through the use of a range of methods, including conventional in vitro fertilization (IVF; the combination of one oocyte with sperm outside the woman’s body) and intracytoplasmic sperm injection (ICSI; a single sperm is injected directly into the egg). The choice is usually based on the sperm parameters. Indeed, poor sperm motility and structure might interfere with fertilization (i.e., crossing the zona pellucida and fusion with the oocyte cytoplasm). The last European Society of Human Reproduction and Embryology (ESHRE) report shows that the ratio of IVF to ICSI has been inverted in the last two decades. Currently, almost 70% of fertilization attempts are done by ICSI [5]. However, the two techniques result in equivalent delivery rates: 29.1% (IVF) and 27.9% (ICSI), according to the last ESHRE report, and 19.2 and 19.3%, respectively, according to the most recent Agence de la Biomédecine report [6].

Male gametes contribute to 50% of the embryo genome. However, while maternal age and the reduction of ovarian reserve are recognized negative prognostic factors for ART [7], the paternal contribution is less well described in the literature.

The aim of this work was to assess the influence of paternal age and sperm quality at ovum pick up on IVF and ICSI outcomes (fertilization rate, early embryo development, rates of blastocyst formation, clinical pregnancy and early miscarriage).

## Methods

### Patients

This retrospective study concerned all couples who underwent IVF or ICSI in our ART centre. In total, 859 IVF and 1632 ICSI cycles were carried out between 1 January 2010 and 31 December 2014. Exclusion criteria were ART cycles using surgically retrieved or frozen sperm, or cycles with pre-implantation genetic diagnosis or using frozen oocytes.

### Sperm preparation

Semen samples were collected by masturbation the day of ovum pick up. Sperm selection for IVF and ICSI treatment was performed by discontinuous PureSperm (Nidacon, Göteborg, Sweden) gradient. Semen samples were layered upon a 40/60/80/90% PureSperm density gradient and centrifuged at 300 g for 15 min. This procedure was followed by a resuspension in 1 mL of sperm culture medium (PureSperm wash, Nidacon, Gothemberg, Sweden) and a second centrifugation at 300 g for 15 min.

### Fertilization technique

The fertilization technique (IVF or ICSI) was chosen during the consultation with the clinical biologist in function of the semen analysis results (WHO 2010 guidelines, [8]).

At ovum pick up, a new semen analysis was systematically performed according to the WHO recommendations. On the basis of its results, the fertilization technique could still be changed. As the morphology analysis was not repeated on that day, the percentage of atypical forms was not included in our criteria of confirmation or modification of the fertilization technique. At least 10% of spermatozoa needed to show rapid progressive motility (formerly, type a motility) before preparation for IVF. After sperm preparation, the volume needed to obtain about 5,000 spermatozoa with normal rapid progressive motility was calculated by considering arbitrarily 50% of teratozoospermia in all samples. This volume was then put in contact, using a micropipette, with the oocyte collected by punction (individual culture in micro-drops). If less than 10% of spermatozoa showed rapid progressive motility, or if a volume higher than 5 μL was needed for each oocyte, IVF was replaced by ICSI.

ICSI could also be directly proposed to couples without male factor in the case of previous low fertilization rate (<30%) or unexplained failure by IVF (after three attempts without pregnancy). During the ICSI procedure, MII oocytes were fixed by a holding pipette and injected with the polar body at 6 or 12 o’clock position.

### Embryo transfer

The embryo transfer strategy used in this study has been previously described [9]. It takes into account the maternal age, her antecedents, the number of previous attempts and the score of the obtained embryos. Embryo selection for transfer was made on the morning of the day 3. One or two embryos were chosen according to their morphology grade. All embryos were evaluated daily with an inverted phase-contrast microscope. They were graded according a simple morphological classification including cell number in relation to the time of development, percentage of cytoplasmic fragmentation and cell symmetry [10]. Four groups were distinguished: excellent (grade 1), good (grade 2), poor (grade 3) and very poor quality embryo (grade 4). When the woman was younger than 36 years, at her first attempt, without major male factor and with at least three high-score embryos at day 3 (grade one or two), one single embryo was transferred at day 5 post-fertilization. In the other cases, one or two embryos were transferred at day 3 and the other embryos were further cultured and then frozen. Only embryos showing development arrest between day 2 and 3 or major cell division defects (<4 cells at day 3) were not further cultured.

During the first three days of development, embryos were cultured individually in 30 μL drops of G1.5TM PLUS (Vitrolife, Göteborg, Sweden) and then they were transferred in a reduced volume of 10 μL of G2.5TM PLUS (Vitrolife, Göteborg, Sweden).

### Embryo cryopreservation

At day 5 or 6, blastocysts of adequate quality, according to the classification by Gardner and Schoolcraft [11], were cryopreserved. The freezing criteria were: grade three or higher blastocysts associated with trophectoderm cells of grade A or B.

### Outcomes

The primary outcomes were: blastulation rate (number of blastocysts obtained at day 5 or day 6/number of embryos in prolonged culture at day 3) and clinical pregnancy rate (beta hCG >1000UI/L and/or heart activity visualized by ultrasonography). The secondary outcomes were the fertilization and early miscarriage rates.

### Data analysis

Results were analysed separately in function of the used fertilization technique (IVF or ICSI). For each technique, cycles were divided in sub-groups on the basis of the sperm concentration before preparation (<0.2, from 0.2 to 5, from 5 to 15 and >15 million/ml), rapid progressive motility before preparation (<32 and >32%) and paternal age (from 20 to 29, from 30 to 39, from 40 to 50 and >51 years).

For each sub-group, the mean maternal age, mean number of oocytes used for fertilization, fertilization rate, mean number of embryos at day 2 and 3, mean number of embryos in prolonged culture, mean number of total blastocysts, mean number of useful blastocysts (i.e., transferred or cryopreserved blastocysts), mean number of transferred blastocysts, rate of clinical pregnancy (beta hCG >1000UI/L and/or heart activity visualized by ultrasonography) and rate of early miscarriage were reported.

Unfortunately, some data are missing in our software program, explaining why the total numbers of cycles is different between each table.

### Statistical analysis

All study data were collected using Microsoft Excel, version 2007. Results were expressed as the mean ± standard deviation (SD) or as percentages. Data were compared with the chi-square test and *p* <0.05 was considered statistically significant. Mean values were compared using the Kruskal-Wallis test.

## Results

### Sperm quality, paternal age and IVF results

None of the studied parameters did significantly vary in function of the sperm concentration, although the clinical pregnancy rate was slightly lower in the < 15 M/ml group (28.9 vs 38%). (Table 1).

**Table 1:** Effects of sperm concentration in IVF

Sperm concentration (M/ml)	Conc < 15	Conc > 15	*P* value
Mean maternal age (years **±** SD)	33.60 ± 4.51	34.36 ± 4.76	0.2235
Number of IVF	52	807	
Mean number of inseminated oocytes ± SD	9.54 ± 5.30	9.83 ± 5.46	0.7268
Rate of fertilization (%)	60.08	66.95	0.7381
Mean number of embryos at day 2 ± SD	5.90 ± 4.56	6.84 ± 4.44	0.0702
Mean number of embryos at day 3 ± SD	5.42 ± 3.81	6.14 ± 4.13	0.1911
Mean number of embryos in prolonged culture ± SD	3.69 ± 4.20	3.87 ± 4.02	0.5102
Mean number of obtained blastocysts ± SD	1.94 ± 2.55	2.06 ± 2.59	0.6040
Rate of blastocyst formation (%)	52.6	53.07	0.9107
Mean number of transferred blastocysts ± SD	0.115 ± 0.323	0.104 ± 0.310	0.8808
Mean number of frozen blastocysts ± SD	1.02 ± 1.58	0.990 ± 1.780	0.7155
Rate of useful blastocysts (%)	58.42	53.22	0.0181
Rate of clinical pregnancy (%)	28.89	37.96	0.228
Rate of miscarriage (%)	30.77	21.80	0.3832

*Conc* concentration, *M/ml* million per ml, *SD* standard deviation

Reduced sperm motility (less than 32% of rapidly progressing spermatozoa) affected the fertilization rate (47.98 vs 67.05% in samples with >32% rapidly progressing spermatozoa; *p* <0.0001) and the mean number of embryos obtained at day 2 (5.18 vs 6.82; *p* = 0.03). The mean number of embryos obtained at day 3 also was slightly reduced (p almost significant). Conversely, the blastocyst formation, clinical pregnancy and early miscarriage rates were not affected by reduced sperm motility (Table 2).

**Table 2:** Effects of progressive motility in IVF

Progressive motility (%)	<32	>32	*P* value
Mean maternal age (years **±** SD)	34.68 ± 4,17	34.31 ± 4.76	0.8207
Number of IVF	22	836	
Mean number of inseminated oocytes ± SD	10.14 ± 7.43	9.81 ± 5.39	0.7195
Fertilization rate (%)	47.98	67.05	<0.0001
Mean number of embryos at day 2 ± SD	5.18 ± 4.47	6.82 ± 4.45	0.0263
Mean number of embryos at day 3 ± SD	4.77 ± 4.14	6.14 ± 4.11	0.0746
Mean number of embryos in prolonged culture ± SD	2.64 ± 3.81	3.90 ± 4.04	0.0972
Mean number of obtained blastocysts ± SD	1.59 ± 2.99	2.06 ± 2.58	0.0792
Rate of blastocyst formation (%)	60.34	52.90	0.2601
Mean number of transferred blastocysts ± SD	0.045 ± 0.213	0.106 ± 0.312	0.6314
Mean number of frozen blastocysts ± SD	0.682 ± 1.550	1.000 ± 1.770	0.25563
Rate of useful blastocysts (%)	45.71	53.65	0.3511
Rate of clinical pregnancy (%)	33.30	37.60	0.7138
Rate of miscarriage (%)	50.00	21.60	0.7155

*SD* standard deviation

Moreover, in the paternal age group >50 years, several values were decreased. As the woman’s mean age was very similar (about 37 years) in the paternal age groups 40–50 and >51 years, an independent effect of the paternal age after the age of 50 could be hypothesized. Specifically, fertilization rate, mean number of embryos at day 2 and 3, mean number of embryos for prolonged culture and mean number of obtained blastocysts were significantly reduced in the age group >51 years compared with the other age groups. The rate of clinical pregnancy was also decreased, but not significantly (Table 3).

**Table 3:** Effects of paternal age on IVF

Paternal age (years)	20 < Age < 29	30 < Age < 39	40 < Age < 50	Age > 51	*P* value
Mean maternal age (years **±** SD)	29.39 ± 5.11	34.22 ± 4.18	37.08 ± 3.43	37.00 ± 5.77	<0.0001
Number of IVF	114	524	211	10	
Mean number of inseminated oocytes **±** SD	10.11 ± 5.72	9.99 ± 5.44	9.31 ± 5.26	7.30 ± 6.17	0.1024
Fertilization rate (%)	64.01	67.66	65.99	42.46	<0.0001
Mean number of embryos at day 2 ± SD	6.76 ± 4.29	7.01 ± 4.58	6.38 ± 4.12	3.40 ± 4.67	0.0104
Mean number of embryos at day 3 ± SD	6.13 ± 3.88	6.31 ± 4.17	5.72 ± 4.02	2.90 ± 4.15	0.0048
Mean number of embryos in prolonged culture ± SD	3.51 ± 3.68	4.09 ± 4.16	3.60 ± 3.86	1.60 ± 3.53	0.0408
Mean number of obtained blastocysts ± SD	1.74 ± 2.35	2.25 ± 2.73	1.78 ± 2.30	0.90 ± 2.02	0.0319
Rate of blastocyst formation (%)	49.50	54.97	49.41	56.25	0.0255
Mean number of transferred blastocysts ± SD	0.053 ± 0.224	0.113 ± 0.322	0.118 ± 0.324	0.000 ± 0.000	0,6987
Mean number of frozen blastocysts ± SD	0.667 ± 1.420	1.040 ± 1.830	1.070 ± 1.780	0.700 ± 1.640	0.3081
Rate of useful blastocysts (%)	41.41	51.10	66.93	77.78	<0.0001
Rate of clinical pregnancies (%)	28.60	38.90	39.70	14.30	0.1151
Rate of miscarriages (%)	17.90	18.60	34.40	**NR**	0.3504

*SD* standard deviation, *NR* not reported (missing data)

The absence of blastocyst transfer in the age group >51 years could be explained by the fact that the woman was often older than 36 years, which in our ART centre leads to a transfer at day 3.

### Sperm quality, paternal age and ICSI results

In ICSI, the fertilization and blastulation rates were significantly reduced in the group with sperm concentration lower than 0.2 M/ml compared with the other sperm concentration groups (*p* <0.0001) (Table 4). Conversely, no clear link was found between sperm concentration and proportion of frozen blastocysts. The lower number of transferred blastocysts in the 0.2 M/ml group could be explained by the transfer strategy used in our ART centre. Indeed, in the case of severe oligospermia, the transfer of two embryos at day 3 is favoured relative to the transfer at day 5. Like for IVF, the clinical pregnancy and early miscarriage rates were not affected by the sperm concentration.

**Table 4:** Effects of sperm concentration in ICSI

Sperm concentration (M/ml)	Conc < 0.2	0.2 < Conc < 5	5 < Conc < 15	Conc > 15	*P* value
Mean maternal age (years **±** SD)	32.97 ± 5.05	31.98 ± 5.11	32.81 ± 5.05	33.94 ± 4.83	0.6257
Number of ICSI	282	373	326	651	
Mean number of injected oocytes **±** SD	8.04 ± 4.52	8.50 ± 4.80	7.81 ± 4.29	7.28 ± 4.24	0.0007
Fertilization rate (%)	58.29	61.87	65.30	68.95	<0.0001
Mean number of embryos at day 2 ± SD	4.40 ± 3.30	5.04 ± 3.70	4.97 ± 3.56	4.81 ± 3.55	0.1515
Mean number of embryos at day 3 ± SD	4.03 ± 3.39	4.21 ± 3.51	4.25 ± 3.30	4.17 ± 3.33	0.7636
Mean number of embryos in prolonged culture ± SD	2.15 ± 2.93	2.78 ± 3.50	2.77 ± 3.43	2.73 ± 3.23	0.0562
Mean number of obtained blastocysts ± SD	0.84 ± 1.63	1.32 ± 2.15	1.44 ± 2.10	1.36 ± 2.07	0.0014
Rate of blastocyst formation (%)	39.17	47.49	51.88	49.69	<0.0001
Mean number of transferred blastocysts ± SD	0.035 ± 0.185	0.042 ± 0.228	0.092 ± 0.300	0.093 ± 0.307	0.3593
Mean number of frozen blastocysts ± SD	0.344 ± 0.980	0.592 ± 1.230	0.610 ± 1.400	0.516 ± 1.210	0.1113
Rate of useful blastocysts (%)	45.155	48.07	48.93	44.96	0.4562
Rate of clinical pregnancies (%)	35.30	41.30	39.60	37.60	0.4980
Rate of miscarriages (%)	22.70	9.60	14.90	21.00	0.1572

*Conc* concentration, *M/ml* million per ml, *SD*, standard deviation

Surprisingly, the fertilization rate was significantly reduced in the group with <32% rapidly progressing spermatozoa (64.82 vs 67.03% in samples with >32% rapidly progressing spermatozoa; *p* <0.013). Conversely, motility did not affect the blastulation, clinical pregnancy and early miscarriage rates (Table 5).

**Table 5:** Effects of progressive motility in ICSI

Progressive motility (%)	<32	>32	*P* value
Mean maternal age (years **±** SD)	33.02 ± 5.13	33.54 ± 4.90	0.0601
Number of ICSI	852	657	
Mean number of injected oocytes **±** SD	8.01 ± 4.48	7.42 ± 4.38	0.0041
Fertilization rate (%)	64.82	67.03	0.013
Mean number of embryos at day 2 ± SD	4.97 ± 3.64	4.82 ± 3.49	0.5315
Mean number of embryos at day 3 ± SD	4.26 ± 3.45	4.16 ± 3.32	0.6800
Mean number of embryos in prolonged culture ± SD	2.75 ± 3.35	2.71 ± 3.26	0.8990
Mean number of obtained blastocysts ± SD	1.37 ± 2.09	1.28 ± 2.02	0.5681
Rate of blastocyst formation (%)	49.76	47.47	0.1441
Mean number of transferred blastocysts ± SD	0.083 ± 0.297	0.067 ± 0.256	0.6823
Mean number of frozen blastocysts ± SD	0.548 ± 1.200	0.530 ± 1.270	0.6363
Rate of useful blastocysts (%)	46.14	46.44	0.8923
Rate of clinical pregnancies (%)	39.50	38.60	0.7272
Rate of miscarriages (%)	17.10	15.30	0.6893

*SD* standard deviation

Finally, the pregnancy rate decreased significantly and the early miscarriage rate tended to be higher in the oldest paternal age group (>51 years) (woman older than 36 years of age) (Table 6).

**Table 6:** Effects of paternal age in ICSI

Paternal age (years)	2 0 < Age < 29	30 < Age < 39	40 < Age < 50	Age > 51	*P* value
Mean maternal age (years **±** SD)	27.54 ± 4.55	32.69 ± 4.12	36.72 ± 3.95	36.53 ± 4.90	<0.0001
Number of ICSI	226	949	410	47	
Mean number of injected oocytes **±** SD	8.69 ± 4.27	7.89 ± 4.53	7.15 ± 4.36	7.32 ± 3.82	<0.0001
Fertilization rate (%)	61.05	65.20	64.57	70.35	0.0007
Mean number of embryos at day 2 ± SD	5.11 ± 3.58	4.92 ± 3.60	4.46 ± 3.45	4.74 ± 3.09	0.0400
Mean number of embryos at day 3 ± SD	4.24 ± 3.28	4.27 ± 3.48	3.88 ± 3.18	4.32 ± 3.30	0.3273
Mean number of embryos in prolonged culture ± SD	2.89 ± 3.51	2.73 ± 3.29	2.35 ± 3.20	2.45 ± 2.86	0.0821
Mean number of obtained blastocysts ± SD	1.46 ± 2.39	1.31 ± 2.00	1.10 ± 1.91	1.13 ± 1.94	0.1376
Rate of blastocyst formation (%)	50.38	48.11	46.99	46.09	0.5682
Mean number of transferred blastocysts ± SD	0.044 ± 0.227	0.077 ± 0.282	0.076 ± 0.274	0.064 ± 0.247	0.8895
Mean number of frozen blastocysts ± SD	0.619 ± 1.300	0.544 ± 1.170	0.410 ± 1.250	0.617 ± 1.410	0.0069
Rate of useful blastocysts (%)	45.59	47.27	43.93	60.38	0.1271
Rate of clinical pregnancies (%)	41.50	41.00	32.10	28.20	0.0162
Rate of miscarriages (%)	17.40	14.60	20.70	45.50	0.3510

*SD* standard deviation

## Discussion

The aim of this retrospective study was to assess the effect of paternal age and of sperm concentration and progressive motility before preparation on the blastulation and clinical pregnancy rates.

In IVF, progressive motility lower than 32% affected significantly the fertilization rate (*p* <0.0001), but not the blastulation, clinical pregnancy and early miscarriage rates. This is in agreement with the work by Chen et al. [12] who did not find any significant difference concerning sperm concentration, total motility, progressive motility and rapid progressive motility between the clinical pregnancy and non-pregnancy groups. Differently from our study where sperm analysis was performed on fertilization day, in the study by Chen et al., sperm was analysed in the 6 months before the attempt.

In ICSI, sperm concentration before preparation did not affect the cycle outcomes (clinical pregnancy or early spontaneous miscarriage). Similarly, rapid progressive motility (< or >32%) did not affect the blastocyst formation, clinical pregnancy and early miscarriage rates. The effect of sperm concentration and rapid progressive motility on the fertilization rate with ICSI (Table 5) could be explained by the bias linked to the association between asthenozoospermia and oligozoospermia. Borges et al. [13] reported that sperm concentration before preparation influences the fertilization rate in ICSI (OR 3.994, *p* = 0.015), but not the rate of blastocyst formation. However, in this study, patients were classified only in two groups (concentration > or <15 M/ml), which did not allow highlighting the effect of severe oligozoospermia on the rate of blastocyst formation found in our study (Table 4).

In our analysis, we did not take into account the percentage of normal forms on fertilization day. This bias is, however, limited because the literature is not unanimous on the effect of teratozoospermia on the outcome of ART attempts. Zhu et al. [14] suggested that isolated teratozoospermia (<4% of normal forms according to the Kruger’s classification [15]) justifies the use of ICSI versus IVF to improve the fertilization rate. Conversely, according to Lockwood et al. [16], teratozoospermia is not a parameter to be taken into account for the choice of ART technique and allows even intra-uterine inseminations. These results confirm those reported by Fan et al. [17]. Many other groups also previously demonstrated the absence of effect of isolated teratozoospermia on IVF results [18–21].

In our study, sperm concentration lower than 0.2 M/ml was the only sperm-related factor that influenced negatively the blastocyst formation rate after ICSI. This is in agreement with the study by Miller and Smith [22] who also found a higher blastulation rate in IVF than in ICSI, which was performed only in the case of reduced sperm motility or of 4 ≤ normal forms. In the same study, increasing paternal age influenced the blastocyst formation rate, independently of the used fertilization technique. We found a similar effect when paternal age was higher than 50 years, but only in the IVF subgroup. In our study, rapid progressive motility did not have any effect on the blastulation rate (both techniques), differently from what reported previously [22, 23].

The effect of severe oligozoospermia on the fertilization and blastulation rates after ICSI could be partially explained by an elevated DNA fragmentation rate (i.e., DNA strand breaks). Many studies have tried to determine the effect of DNA fragmentation on the fertilization, pregnancy and spontaneous miscarriage rates. At low level, these breakages are repaired in the oocyte after fertilization. Beyond a certain level, they cannot be efficiently repaired to allow normal embryo development. Many studies have observed the absence of links between the DNA fragmentation index (DFI) and the quality of embryos generated with ICSI [24–30]. Moreover, according to Evenson et al. [31], improving sperm chromatin structure during ART is not associated with higher fertilization and embryo quality rates. The absence of correlation between DFI and embryo quality could be partially explained by the fact that better quality spermatozoa are chosen for ICSI [30]. However, other studies have highlighted the existence of an association between the level of sperm DNA fragmentation and degree of embryo fragmentation after ICSI [32–39]. Guérin et al. [40] never obtained a successful pregnancy, when sperm DFI was higher than 45%, whatever the ART technique used. Larson et al. [41] found that if more than 27% of spermatozoa show DNA denaturation, no pregnancy can be obtained after ICSI. For Simon et al. [42], increased sperm DNA fragmentation is associated with abnormal protamination and results in lower fertilization rates, poorer embryo quality and reduced pregnancy rates.

New approaches are needed for sperm selection. A very interesting study by Muratori et al. [43] provides the evidence that the density gradient centrifugation procedure produces an increase in sperm DNA fragmentation in some subjects undergoing IVF/ICSI, who then show a much lower probability of pregnancy, raising concerns about the safety of this selection procedure. Alternative sperm selection strategies are recommended for those patients.

Unfortunately, we did not record male body mass index (BMI) in our study. This is obviously a limitation for our results interpretation as more and more studies have highlighted to role of BMI of men on fertility issues. In 2013, Anifandis et al. [44] showed that even if male BMI did not correlate with sperm parameters, the groups with the highest BMI had the lowest embryo quality. Moreover, the combination of BMI and age of both men and women had a negative effect on pregnancy rate. A more recent systematic review with thirty papers included [45] confirm these results. Men obesity does not influence conventional sperm parameters but is associated with reduction of live birth rate per cycle and an increase of DNA fragmentation.

The effects of paternal age are difficult to interpret due to the maternal age bias. Indeed, paternal age is strictly correlated with that of the female partner. Our univariate analysis did not allow determining the incidence of paternal age «alone» on the different parameters under study. Nevertheless, our results suggest that from the age of 51 years, paternal age negatively affects the rates of blastocyst formation (IVF) and of clinical pregnancy (ICSI). For both techniques, the rate of early miscarriage was slightly higher (but not significantly) in the age group >51 years. The pregnancy rate reduction associated with increasing paternal age has been already described by Hassan and Killik [46] and De La Rochebrochard and Thonneau [47]). Increasing paternal age also has an effect on the rate of early miscarriages. De La Rochebrochard et al. [48] reported that in the case of maternal age over 35 years and paternal age over 40 years, the risk of early miscarriage is increased. For Kleinhaus [49], this risk is present from the age of 40 years, also after adjustment for maternal age (OR 1.6 vs <25 years). Finally, Slama [50] found that the risk of early miscarriage is higher starting from the paternal age of 35 years. Finally, there is probably a cumulative effect of increasing paternal and maternal age. According to our results, we consider as threshold ages 37 years for women and 51 for men.

## Conclusion

This study shows that some male factors are significantly associated with ART outcomes. Independently of the used technique (IVF or ICSI), sperm rapid progressive motility affect the fertilization rate. In ICSI, oligospermia affect the fertilization rate and severe oligospermia (<0.2 M/ml) causes also a reduction of the blastulation rate. Increasing paternal age (>51 years) negatively influences the rate of clinical pregnancy when the woman is older than 37 years. These results allow adjusting the fertilization and embryo transfer strategies in our ART centre. For severe oligospermia, it should be better to propose a transfer at the cleaved embryo stage because the blastocyst formation rate is significantly reduced. Finally, different promising research avenues, described in the recent study by Bounartzi et al. [51], should be explored, such as the quantification of free sperm DNA to optimize the selection of male gametes and thus improving ART results.

## References

[1] Bouillon C, Fauque P (2013). Follow-up of children conceived by assisted reproductive technologies. Arch Pediatr.

[2] Ray A, Shah A, Gudi A, Homburg R (2012). Unexplained infertility: an update and review of practice. Reprod Biomed Online.

[3] Cooper TG, Noonan E, von Eckardstein S, Auger J, Baker HW, Behre HM (2010). WHO reference values for human semen characteristics. Hum Reprod Update.

[4] Rolland M, LeMoal J, Wagner V, Royère D, De Mouzon J (2013). Decline in semen concentration and morphology in a sample of 26 609 men close to general population between 1989 and 2005 in France. Hum Reprod.

[5] Kupka MS, D’Hooghe T, Ferraretti AP, de Mouzon J, Erb K, Castilla JA (2016). Assisted reproductive technology in Europe, 2011: results generated from European registers by ESHREdoi: 10.1093/humrep/des415European IVF-monitoring consortium (EIM); European society of *Human Peprod* and embryology (ESHRE). Hum Reprod.

[6] Rapport d’activités de l’Agence de Biomédecine. Available at: URL: http://www.agence-biomedecine.fr/.

[7] van Loendersloot LL, van Wely M, Limpens J, Bossuyt PM, Repping S, van der Veen F (2010). Predictive factors in in vitro fertilization (IVF): a systematic review and meta-analysis. Hum Reprod Update.

[8] WHO guidelines. Available at: URL: http://www.who.int/reproductivehealth/publications/infertility/9789241547789/en/.

[9] Gala A, Ferrières A, Assou S, Monforte M, Bringer-Deutsch S, Vintejoux E (2014). Effects of artificial shrinkage prior to vitrification in a closed system: a randomized controlled trial. Gynecol Obstet Fertil.

[10] Alpha Scientists in Reproductive Medicine and ESI (2011). The Istanbul consensus workshop on embryo assessment: proceedings of an expert meeting. Hum Reprod.

[11] Gardner DK, Schoolcraft WB (1999). In vitro culture of human blastocysts. Reprod Certain Fertil Genet Beyond.

[12] Chen X, Zhang W, Luo Y (2009). Predictive value of semen parameters in in vitro fertilisation pregnancy outcome. Andrologia.

[13] Borges E, Setti AS, Braga DP, Figueira RC, Iaconelli A (2016). Total motile sperm count has a superior predictive value over the WHO 2010 cut-off values for the outcomes of intracytoplasmic sperm injection cycles. Andrology.

[14] Zhu Y, Wu QF, Zhou XJ, Xin CL, Ling G, Lu GX (2013). ICSI improves fertilization in isolated teratozoospermic men: a study with strictly controlled external factors and WHO-5 standard. Syst Biol Reprod Med.

[15] Kruger TF, Menkveld R, Stander FS, Lombard CJ, Van der Merwe JP, van Zyl JA, Smith K (1986). Sperm morphologic features as a prognostic factor in in vitro fertilization. Fertil Steril.

[16] Lockwood GM, Deveneau NE, Shridharani AN, Strawn EY, Sandlow JI (2015). Isolated abnormal strict morphology is not a contraindication for intrauterine insemination. Andrology.

[17] Fan W, Li SW, Li L, Huang Z, Ma Q, Wang Y, Xiao Z (2012). Outcome of conventional IVF and ICSI on sibling oocytes in the case of isolated teratozoospermia. J Assist Reprod Genet.

[18] Figueiredo H, Tavares A, Ferras L, Couceiro A, Chaves I (1996). Isolated teratozoospermia and in vitro fertilization. J Assist Reprod Genet.

[19] Nallella KP, Sharma RK, Aziz N, Argawal A (2006). Significance of sperm characteristics in the evaluation of male infertility. Fertil Steril.

[20] Keegan BR, Barton S, Sanchez X, Berkeley AS, Krey LC, Grifo J (2007). Isolated teratozoospermia does not affect in vitro fertilization outcome and is not an indication for intracytoplasmic sperm injection. Fertil Steril.

[21] Hotaling JM, Smith JF, Rosen M, Muller CH, Walsh TJ (2011). The relationship between isolated teratozoospermia and clinical pregnancy after in vitro fertilization with or without intracytoplasmic sperm injection: a systematic review and meta-analysis. Fertil Steril.

[22] Miller JE, Smith TT (2001). The effect of intracytoplasmic sperm injection and semen parameters on blastocyst development in vitro. Hum Reprod.

[23] Janny L, Menezo YJ (1994). Evidence for a strong paternal effect on human preimplantation embryo development and blastocyst formation. Mol Reprod Dev.

[24] Lopes S, Sun J, Jurisicova A, Meriano J and Casper RF. Semen deoxyribonucleic acid fragmentation in intracytoplasmic sperm injection. Fertil Steril. 1998b;69:528–532. 10.1016/s0015-0282(97)00536-0 9531891

[25] Shoukir Y, Chardonnens D, Campana A, Sakkas D (1998). Blastocyst development from supernumerary embryos after intracytoplasmic sperm injection: a paternal influence?. Hum Reprod.

[26] Benchaib M, Braun V, Lornage J, Hadj S, Salle B, Lejeune H (2003). Sperm DNA fragmentation decreases the pregnancy rate in an assisted reproductive technique. Hum Reprod.

[27] Larson-Cook KL, Brannian JD, Hansen KA, Kasperson KM, Aamold ET, Evenson DP (2003). Relationship between the outcomes of assisted reproductive techniques and sperm DNA fragmentation as measured by the sperm chromatin structure assay. Fertil Steril.

[28] Huang CC, Lin DPC, Tsao HM, Cheng TC, Miu CH, Lee MS (2005). Sperm DNA fragmentation negatively correlates with velocity and fertilization rates but might not affect pregnancy rates. Fertil Steril.

[29] Bungum M, Humaidan P, Axmon A, Spano M, Bungum L, Erenpreiss J (2007). Sperm DNA integrity assessment in prediction of assisted reproduction technology outcome. Hum Reprod.

[30] Sadeghi MR, Lakpour N, Heidari-Vala H, Hodjat M, Amirjannati N, Hossaini JH (2011). Relationship between sperm chromatin status and ICSI outcome in men with obstructive azoospermia and unexplained infertile normozoospermia. Rom J Morphol Embryol.

[31] Evenson DP, Larson KL, Jost LK (2002). Sperm chromatin structure assay: its clinical use for detecting sperm DNA fragmentation in male infertility and comparisons with other techniques. J Androl.

[32] Aboulghar MA, Mansour RT, Serour GI, Fahmy I, Kamal A, Tawab NA (1997). Fertilization and pregnancy rates after intracytoplasmic sperm injection using ejaculate semen and surgically retrieved sperm. Fertil Steril.

[33] Brinkworth MH (2000). Paternal transmission of genetic damage: findings in animals and humans. Int J Androl.

[34] Virant-Klun I, Tomazevic T, Meden-Vrtovec H (2002). Sperm single-stranded DNA, detected by acridine orange staining, reduces fertilization and quality of ICSI-derived embryos. J Assist Reprod Genet.

[35] Perreault SD (2003). Distinguishing between fertilization failure and early pregnancy loss when identifying male-mediated adverse pregnancy outcomes. Adv Exp Med Biol.

[36] Saleh RA, Agarwal A, Sharma RK, Said TM, Sikka SC, Thomas AJ (2003). Negative effects of increased sperm DNA damage in relation to seminal oxidative stress in men with idiopathic and male factor infertility. Fertil Steril.

[37] Borini A, Tarozzi N, Bizzaro D, Bonu MA, Fava L, Flamigni C (2006). Sperm DNA fragmentation: paternal effect on early post-implantation embryo development in ART. Hum Reprod.

[38] Muriel L, Garrido N, Fernández JL, Remohí J, Pellicer A, de los Santos MJ (2006). Value of the sperm deoxyribonucleic acid fragmentation level, as measured by the sperm chromatin dispersion test, in the outcome of in vitro fertilization and intracytoplasmic sperm injection. Fertil Steril.

[39] Babazadeh Z, Razavi S, Tavalaee M, Deemeth MR, Shahidi M, Nasr-Esfahani MH (2010). Sperm DNA damage and its relation with leukocyte DNA damage. Reprod Toxicol.

[40] Guérin P, Matillon C, Bleau G, Lévy R, Ménézo Y (2005). Impact of sperm DNA fragmentation on ART outcome. Gynecol Obstet Fertil.

[41] Larson KL, DeJonge CJ, Barnes AM, Jost LK, Evenson DP (2000). Sperm chromatin structure assay parameters as predictors of failed pregnancy following assisted reproductive techniques. Hum Reprod.

[42] Simon L, Castillo J, Oliva R, Lewis SE (2011). Relationships between human sperm protamines, DNA damage and assisted reproduction outcomes. Reprod Biomed Online.

[43] Muratori M, Tarozzi N, Cambi M, Boni L, Iorio AL, Passaro C (2016). A variation of DNA fragmentation levels during density gradient sperm selection for assisted reproduction techniques: a possible New male predictive parameter of pregnancy?. Medicine (Baltimore).

[44] Anifandis G, Dafopoulos K, Messini CI, Polyzos N, Messinis IE (2013). The BMI of men and not sperm parameters impact on embryo quality and the IVF outcome. Andrology.

[45] Campbell JM, Lane M, Owens JA, Bakos HW (2015). Paternal obesity negatively affects male fertility and assisted reproduction outcomes: a systematic review and meta-analysis. Reprod Biomed Online.

[46] Hassan MA, Killick SR (2003). Effect of male age on fertility: evidence for the decline in male fertility with increasing age. Fertil Steril.

[47] De La Rochebrochard E, Thonneau P (2003). Paternal age > or = 40 years: an important risk factor for infertility. Am J Obstet Gynecol.

[48] De La Rochebrochard E, McElreavey K, Thonneau P (2002). Paternal age over 40 years: the “amber light” in the reproductive life of men?. J Androl.

[49] Kleinhaus K, Perrin M, Friedlander Y, Paltiel O, Malaspina D, Harlap S (2006). Paternal age and spontaneous abortion. Obstet Gynecol.

[50] Slama R, Bouyer J, Windham G, Fenster L, Werwatz A, Swan SH (2005). Influence of paternal age on the risk of spontaneous abortion. J Epidemiol.

[51] Bounartzi T, Dafopoulos K, Anifandis G, Messini CI, Koutsonikou C, Kouris S (2016). Pregnancy prediction by free sperm DNA and sperm DNA fragmentation in semen specimens of IVF/ICSI-ET patients. Hum Fertil.

